# Contract cheating by STEM students through a file sharing website: a Covid-19 pandemic perspective

**DOI:** 10.1007/s40979-021-00070-0

**Published:** 2021-02-04

**Authors:** Thomas Lancaster, Codrin Cotarlan

**Affiliations:** Department of Computing, Imperial College London, London, UK

**Keywords:** Chegg, Academic integrity, Contract cheating, File sharing, Academic misconduct, Online exams, Assessment, Covid-19, Pandemic

## Abstract

Students are using file sharing sites to breach academic integrity in light of the Covid-19 pandemic. This paper analyses the use of one such site, Chegg, which offers “homework help” and other academic services to students. Chegg is often presented as a file sharing site in the academic literature, but that is just one of many ways in which it can be used. As this paper demonstrates, Chegg can and is used for contract cheating This is despite the apparent existence of an Honour Code on Chegg which asks students not to breach academic integrity. With pandemic led safety considerations leading to increased online teaching and assessment, the paper analyses data relating to how Chegg is used by students in five STEM subjects, namely Computer Science, Mechanical Engineering, Electrical Engineering, Physics and Chemistry. The results show that students are using Chegg to request exam style questions. They demonstrate that contract cheating requests can be put live and answered within the short duration of an examination. The number of student requests posted for these five subjects increased by 196.25% comparing the time period April 2019 to August 2019 with the period April 2020 to August 2020. This increase corresponds with the time when many courses moved to be delivered and assessed online. The growing number of requests indicates that students are using Chegg for assessment and exam help frequently and in a way that is not considered permissible by universities. The paper concludes by recommending that academic institutions put interventions in place to minimise the risk to educational standards posed by sites such as Chegg, particularly since increased online teaching and assessment may continue after the pandemic.

## Introduction

This paper reports on the growth of how a single market leading file sharing website has been used for contract cheating purposes. The period of growth coincides with the Covid-19 pandemic and the associated necessary increase in online teaching and assessment within education.

Rogerson and Basanta (2016) define file sharing as being “*when academic lecture materials, notes, assessment tasks, answers, and responses are shared, swapped, and traded over Internet-based sites in fee, free, or barter (credit/exchange) arrangements*”. Although student collaboration is often encouraged within an educational setting, a challenge with file sharing comes when students share files owned by a university with commercial providers. These providers then sell materials which they have no ownership claim over to other students. Often a barter system is used, with other students encouraged to upload more materials so that they can themselves get access to other resources and answers. As well as breaching copyright and instructor rights of intellectual property, this also has commercial implications for educational providers. Dixon and George (2020) review the value of content on a single file sharing site and estimate that the materials for a frequently taught course at a single university cost $70,000 United States Dollars (USD) to produce. They estimate the value of a programme refreshed every 5 years as being $3.5 million USD over that time period, a sizeable value potentially lost from educational providers.

The use of file sharing sites to breach academic integrity has received little attention in the academic literature. Various terms are used to be describe file sharing sites, including in the literature and by the sites themselves. These terms include crowd sourcing sites, study aid sites and peer-to-peer platforms. Example file sharing sites include OneClass (2020), Chegg (2020), Course Hero (2020) and Thinkswap (2020). This is not a complete list and file sharing sites exist aimed at only at individual universities and in several languages. In many ways, these sites can be considered as an extension of traditional fraternity-based archives of previous assignment solutions, designed to give fraternity members an unfair advantage.

Although file sharing sites are already ethically questionable, a further challenge comes when they can also be used for commercial contract cheating purposes. Contract cheating, originally discussed by Clarke and Lancaster (2006), takes place when a student employs a third party to complete assessed work for them. Many file sharing sites contain a section of the site which can be used for contract cheating purposes, often billed as providing “homework help”. Although traditionally contract cheating requests are sold only to a single student, file sharing sites often operate with a variant strategy where answers can be made available to many students, once the appropriate payment has been made or bartering has concluded.

This paper considers how contract cheating takes place on the market leading file sharing site Chegg (2020). It makes reference to the volume of requests made and answers supplied pre and post Covid-19. The pandemic has seen the movement of teaching and assessment online, often made with little time for the revised method of provision to have been planned in advance or for academic integrity safeguards to be put into place. Where students have previously been taught face-to-face, activities such as in-person lectures, tutorials, assessments and exams have been replaced by virtual alternatives. The unsupervised nature of assessment, including exams, may mean that students have had increased temptation to cheat or may have felt that the support they would usually have available was not there.

The paper first discusses the relevant literature surrounding academic integrity, contract cheating and online exams in more detail. Online teaching and assessment are not in themselves new, even though changes to assessment due to Covid-19 may have made this more prominent. The Chegg file sharing site is further discussed, with reference to how this can be used for contract cheating purposes. The paper provides a quantitative analysis of how Chegg is used for contract cheating within a selection of Science, Technology, Engineering and Mathematics (STEM) subjects, offering an analysis over a two-year period with reference to pre and post Covid-19 provision. The paper concludes by recommending that the sector works to address contract cheating through file sharing sites particularly as this relates to Covid-19.

## Background

Many forms of assessment are susceptible to contract cheating. The literature in this field has revealed that an aggressive industry exists, aiming to implore students to cheat (Ellis et al. 2018; Lancaster 2020a). Contract cheating solutions can be purchased cheaply by students, often from writers operating in economic surroundings where incomes are typically low (Lancaster 2020b). Contract cheating solutions can also be provided quickly (Wallace and Newton 2014). This includes providing them within the limited time available for a standard online examination.

Where courses are taught online, contract cheating is a particular risk. Lancaster and Clarke (2014) reviewed how students at online universities were using a site that nominally stated it connected students with tutors for contract cheating. They found the bulk of requests were from the United States, mainly from the Business and Computing subject areas. Students themselves working as academic integrity partners with educators have subsequently begun to use the term “toxic tutors” to describe those individuals advertising themselves as providing help, but actually there to do work for students (ICAI 2020). Students have advised their peers to carefully consider the services tutoring services say they offer and to choose providers with care to avoid accidentally breaching academic integrity.

Examinations themselves have also been found to be susceptible to contract cheating. Lancaster and Clarke (2017) identified a wide range of sites that could be used to provide students with unauthorised exam assistance, including tutorial sites. Where exams are online, remote proctoring services that use cameras to check the activities of students have been suggested as possible solutions. However, experts have warned about the dangers of such an approach. Eaton and Turner (2020) conducted a rapid review into literature on academic integrity relating to Covid-19. They identified that students felt they were suffering from stress and anxiety, particularly when remote proctoring solutions were used to preserve academic integrity. However, when students are not monitored during examinations, they may be able to turn to file sharing websites to request contract cheating solutions. Although further research in this field is necessary, this does illustrate the trade-off between the need to protect the value of academic awards, but to still ensure that students feel supported and do not need to use suspect providers of services from outside their own academic institution.

Research has shown that cheating is more likely to occur during online exams than on-site exams. From a survey of accounting students, King et al. (2009) found that students believed that cheating in online exams was easier than cheating in exams held in person. They also noted that students said they would be less likely to cheat if instructors specifically told them that this was not allowed.

The solutions to exam integrity breaches through file sharing websites need to be considered. Cluskey Jr et al. (2011) suggested changing the questions for online exams every time they run. This would prevent standard answers being already available on file sharing websites, but that, in itself, would not seem to be a solution to contract cheating.

Clark et al. (2020) have recommended specific solutions to online exam integrity in light of Covid-19. They found that contract cheating was occurring in online chemistry exams and suggest watermarking exam materials to make them more difficult to share with contract cheating providers. They also recommend the use of unique data sets for individual students to work on. This means that if questions are placed on a visible file sharing site, the student with that data set allocated to them can be traced. Even where this is not a viable solution, it can be possible to detect contract cheating, including answers obtained from file sharing sites. Rogerson (2017) provided indicators for assessors to look out for, including citation and referencing irregularities, as well as the use of inconsistent language.

Quantitative academic research relating to the use of file sharing sites by students is limited. In a study conducted pre Covid-19, Bretag et al. (2019) surveyed students at Australian educational institutions and asked them about their file sharing tendencies. They found that 15.3% said they had brought or traded notes and 27.2% had provided assignments to other students. They found that 2.4% of students said they had received assistance during examinations. Although these results are not specifically linked to file sharing websites, they do suggest that many students could be encouraged to use sites like these.

Grams (2011a, 2011b) tested the hypothesis that students would perform better if they had legitimate and approved access to the materials from a file sharing site. Grams noted that, despite students believing access would positively increase their grades, there was no noticeable difference between students who were granted access to such a site for a year compared to other students. Instructors generally had a negative opinion about the use of such sites. Grams also noted that the students preferred to learn from textbook solutions over those provided through the file sharing site being examined. Van de Sande (2011) independently reviewed the quality of solutions on such a site and found that 56% of them were not as good as those found in instructor solution manuals.

Ardid et al. (2015) found no difference in the results students received when taking in-person and online exams, provided both types of exams were proctored. However, when students took the exam online and it was not proctored, students received higher marks than in a proctored situation. A similar result was found by Nizam et al. (2020) in research conducted during Covid-19. They also found that students obtained higher marks in unproctored exams had higher marks than in proctored exams. This may be due to access to students having access to the contract cheating industry in such a situation.

If contract cheating opportunities are growing as a result of Covid-19, there are other risks to consider. Yorke et al. (2020) have warned how students are at risk of blackmail, both during their course and following its completion. They have also shown that most students are unaware of the risks of using contract cheating providers.

## Methodology

This study reviews the available of contract cheating solutions through the file sharing site Chegg (2020). Amongst other services offered, Chegg provides a homework help section, where people can post problems and request full solutions. These requests might include homework questions, textbook problems, assignments and exam questions. Free users can view questions, but not post them. Subscribers can post up to 20 questions per month and view all answers. Answers can be provided by regular Chegg users, but also by a group that Chegg has certified as experts. Not every question receives an answer and questions can receive more than one answer. The question archive is split into subject categories making it possible to see how students self-classified their requests. This archive can also be searched by keyword.

For this study, the Chegg archive of homework help questions was examined for five subjects in the STEM grouping to provide an indication of how the site was being used. The subjects investigated were Computer Science, Mechanical Engineering, Electrical Engineering, Physics and Chemistry. Quantitative data related to the number of questions posted per subject per day was collected over a two-year period from 1 September 2018 to 31 August 2020. The researchers did not subscribe to the Chegg service to collect this data, which was freely accessible and manually collected. The data was collected directly from the subject level menu on Chegg. The data set provides two complete years of Chegg data for the five subjects, allowing for comparisons between 1 September 2018 to 31 August 2019, with 1 September 2019 to 31 August 2020, dates roughly equivalent to the academic year in many countries.

For simplicity in the remainder of the paper, the period from 1 September 2018 to 31 August 2019 will be referred to as 18/19. The period from 1 September 2019 to 31 August 2020 will be referred to as 19/20.

In addition, a single typical day was selected, consistent for each year. The number of questions that day receiving at least one answer was calculated. This single day comparison was intended to allow researchers to identify if, in the event of the number of questions asked changing, did this affect the number of questions being answered?

### Research methodology limitations

Due to the labour intensive nature of this research, data collection was restricted to a limited range of subjects for a two-year period. The subjects were selected to be STEM based to allow this field to be considered in more depth. The timeline for data collection was considered to allow the impact of Covid-19 to be explored with relation to contract cheating. The research also only considers a single file sharing site, so it is not certain if these results will generalise to other such sites.

## Results and discussion

In total, across the five analysed subjects there were 3,050,372 questions posted during 18/19 and 5,335,770 questions posted between during 19/20. This showed an increase in the number of questions posted of 74.92% from 18/19 to 19/20. The average (mean) number of questions posted in 18/19 was 8357 and in 19/20 was 14,578. Table 1 shows this calculation on a subject basis.

**Table 1:** Questions posted on Chegg in 18/19 and 19/20

	Total number of questions per year	Average number of questions per day	Percentual increase from year 2018–2019 to year 2019–2020
Computer Science 19/20	988,403	2700.55	57.03
Computer Science 18/19	629,402	1724.39	
Physics 19/20	1,130,991	3090.14	77.91
Physics 18/19	635,677	1741.58	
Chemistry 19/20	1,758,165	4803.73	99.53
Chemistry 18/19	881,122	2414.03	
Mechanical Engineering 19/20	71,3243	1948.75	64.49
Mechanical Engineering 18/19	433,599	1187.94	
Electrical Engineering 19/20	744,968	2035.43	58.31
Electrical Engineering 18/19	470,572	1289.24	
All Subjects 19/20	5,335,770	14,578.61	74.92
All Subjects 18/19	3,050,372	8357.18	

The number of questions asked and answered on 1 March each year was also calculated. Eight thousand two hundred seventy-six questions were posted 1 March 2018 across all five subjects. These were answered 89.96% of the time. Thirteen thousand seven hundred nineteen questions were posted on 1 March 2020 and answered 85.57% of the time. Despite the slight drop in the percentage of questions answered, the raw numbers showed a substantial quantitative increase.

Table 2 shows this information at subject level for 1 March each year. Of note is the decrease in the percentage of answered questions in Computer Science, although this is still an increase in numeric terms.

**Table 2:** Answered Questions on 1 March 2019 and 1 March 2020

	Total number of questions on 1 March	Total number of unanswered questions on 1 March	Percentage of answered questions
Computer Science 19/20	2974	1173	60.55
Computer Science 18/19	1852	494	73.32
Physics 19/20	2725	83	96.95
Physics 18/19	1938	67	96.54
Chemistry 19/20	4805	430	91.05
Chemistry 18/19	2347	106	95.48
Mechanical Engineering 19/20	1619	138	91.47
Mechanical Engineering 18/19	958	88	90.81
Electrical Engineering 19/20	1596	155	90.28
Electrical Engineering 18/19	1181	76	93.56
All Subjects 19/20	13,719	1979	85.57
All Subjects 18/19	8276	831	89.95

The average number of questions per day per month was also calculated at a subject level and compared on a month-by-month basis. This showed a striking result. Between September and March, inclusive, the percentage increase never exceeded 32.27% for any of the subjects. Between April and August, inclusive, the smallest increase was 80.08% and the largest increase 343%. Table 3 provides an example of this for Chemistry as monthly averages. The pattern shows a slight increase in March as universities began to move to online teaching, then a much greater increase from April onwards.

**Table 3:** Percentage increase per day per month of Chemistry questions posted between 18/19 and 19/20

	Percentual increase of the average number of questions posted per day per month between Chemistry 18/19 and Chemistry 19/20
September	23.54
October	18.60
November	13.00
December	18.44
January	18.36
February	16.62
March	32.26
April	172.03
May	302.63
June	290.18
July	288.09
August	325.74

**Fig. 1:**
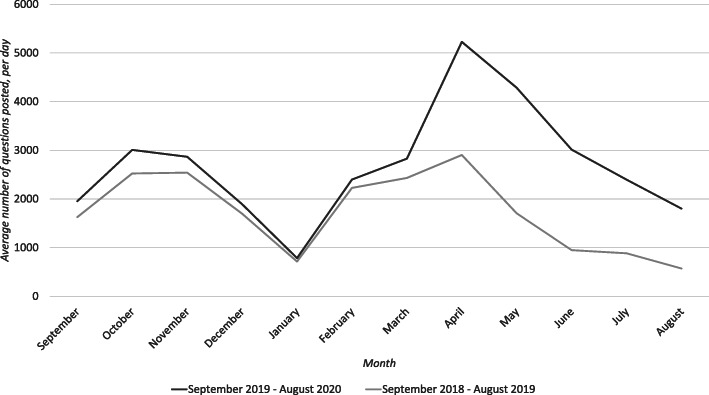
Questions per day per month of Computer Science questions posted between 18/19 and 19/20

**Fig. 2:**
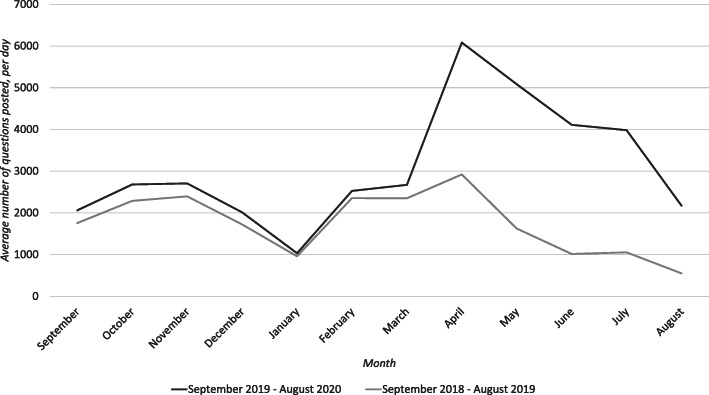
Questions per day per month of Physics questions posted between 18/19 and 19/20

**Fig. 3:**
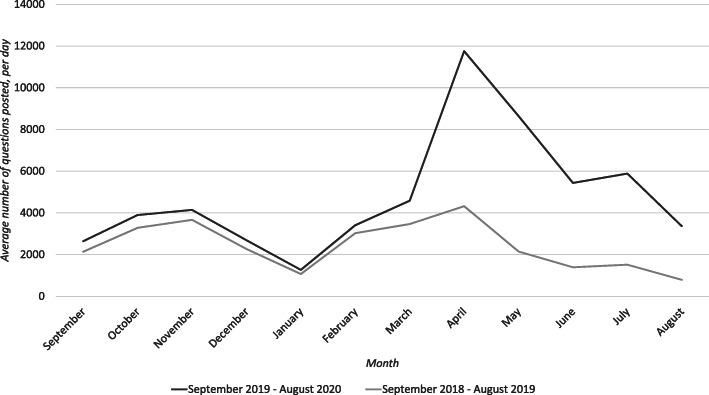
Questions per day per month of Chemistry questions posted between 18/19 and 19/20

**Fig. 4:**
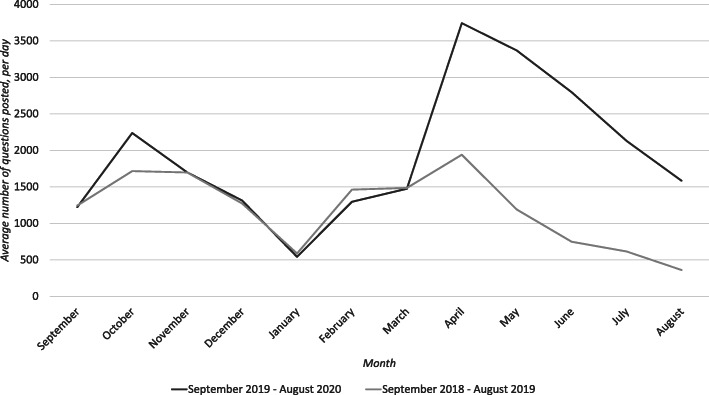
Questions per day per month of Mechanical Engineering questions posted between 18/19 and 19/20

**Fig. 5:**
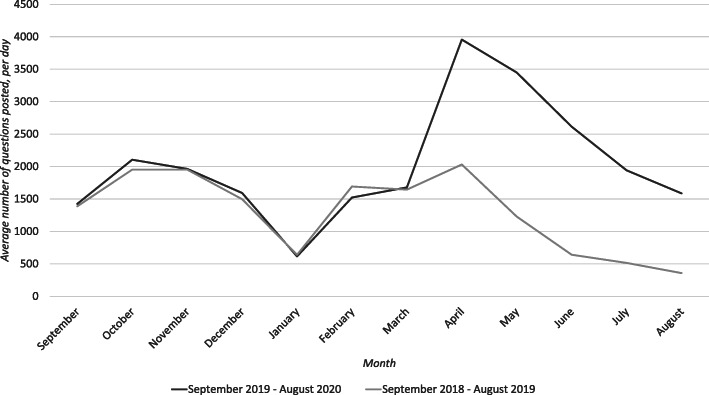
Questions per day per month of Electrical Engineering questions posted between 18/19 and 19/20

Figures 1, 2, 3, 4 and 5 graphically illustrate the average number of questions asked per day per month for each subject across the 18/19 and 19/20 period. Although the numbers differ slightly, the graphs show a largely similar shape. They illustrate two peak request times for contract cheating services each year, with one in April–May and another in October–November. These may link with university assessment periods. The April–May peak for 19/20 is of a greater order of magnitude than that from 18/19.

For example, in Fig. 1 (Computer Science), the maximum peak values in 18/19 in November was 2541 questions and in April was 2903 questions, but in 19/20, the peak in November was 2867 questions and in April this rose to 5228 questions. The graph also shows that the gradient of the slope between the years after April is very similar, with the number of questions posted decreasing each month. It is important to notice that these trends occurred regardless of the subject area. Based on the sample, it looks likely that the same trend will occur in subjects outside those selected for analysis.

**Fig. 6:**
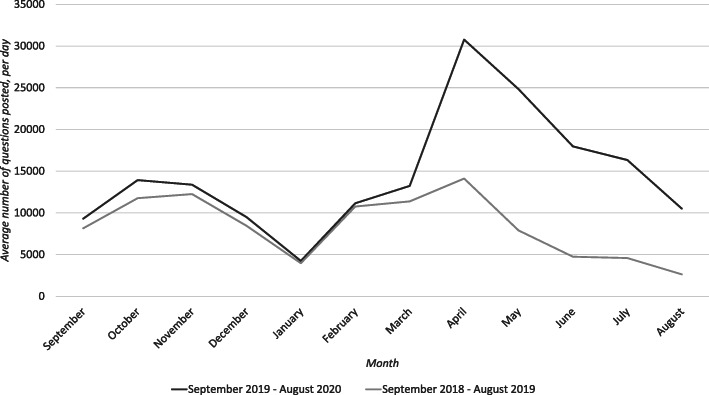
Questions per day per month of questions from all STEM subjects analysed, posted between 18/19 and 19/20

Figure 6 shows similar information to Figs. 1, 2, 3, 4 and 5, only for the five subjects being considered taken as overall totals. The shape of the graph and the overall peaks are again consistent with the idea that the use of Chegg to request answers has increased year-on-year in the post Covid-19 period.

A further analysis considered the average number of questions asked per day in the September to March period and in the April to August period, compared over the 2 years. Between September and March, this showed an overall 12.68% increase between the years of the average number of questions posted per day per month, but between April and August the increase was 196.25%, which is a substantial difference. Furthermore, the average had dropped by 28.92% between the September 2018–March 2019 and April 2019–August 2019 periods, but increased by 87.74% between the September 2019–March 2020 and April 2020–August 2020 periods.

Table 4 details the percentage changes over the two time periods at subject level.

**Table 4:** Subject comparison of the September to March and April to August time periods

	Average number of questions per day	Percentual increase of the average from September–March to April–August	Percentual increase of the average of the previous year
Computer Science September 2019–March 2020	2244.91	48.55	14.97
Computer Science April 2020–August 2020	3334.88		138.97
Computer Science September 2018–March 2019	1961.74	−28.86	
Computer Science April 2019–August 2019	1395.50		
Physics September 2019–March 2020	2238.16	91.05	14.20
Physics April 2020–August 2020	4276.21		199.79
Physics September 2018–March 2019	1969.05	−27.55	
Physics April 2019–August 2019	1426.38		
Chemistry September 2019–March 2020	3229.19	116.64	20.32
Chemistry April 2020–August 2020	6995.73		245.86
Chemistry September 2018–March 2019	2696.47	−24.98	
Chemistry April 2019–August 2019	2022.67		
Mechanical Engineering September 2019–March 2020	1397.31	94.40	4.09
Mechanical Engineering April 2020–August 2020	2716.43		181.43
Mechanical Engineering September 2018–March 2019	1348.69	−28.43	
Mechanical Engineering April 2019–August 2019	965.20		
Electrical Engineering September 2019–March 2020	1556.53	73.59	1.93
Electrical Engineering April 2020–August 2020	2702.12		184.51
Electrical Engineering September 2018–March 2019	1534.26	−38.09	
Electrical Engineering April 2019–August 2019	949.71		
All Subjects September 2019–March 2020	10,666.13	87.74	12.68
All Subjects April 2020–August 2020	20,025.38		196.25
All Subjects September 2018–March 2019	9510.24	−28.92	
All Subjects April 2019–August 2019	6759.48		

Although Table 4 shows only a slight increase in the number of questions posted during the September to March periods, the April to August periods show an overall increase of 196.25%. This indicates that the file sharing site has been used much more during this time. Similar trends were observed across all five subjects. Chegg usage decreased approaching August and September 2019, but increased in the following year, further reinforcing the observation made above.

Chegg do not provide the facility to trace requests directly back to an individual academic provider, or even a country of origin. On occasion it may be possible to work this out, but the way in which data is shown on the site makes this difficult. For example, some posts contain photos or screenshots of a problem that is likely to have been encountered in a standard textbook. Such standard questions could be in use at many institutions.

**Fig. 7:**
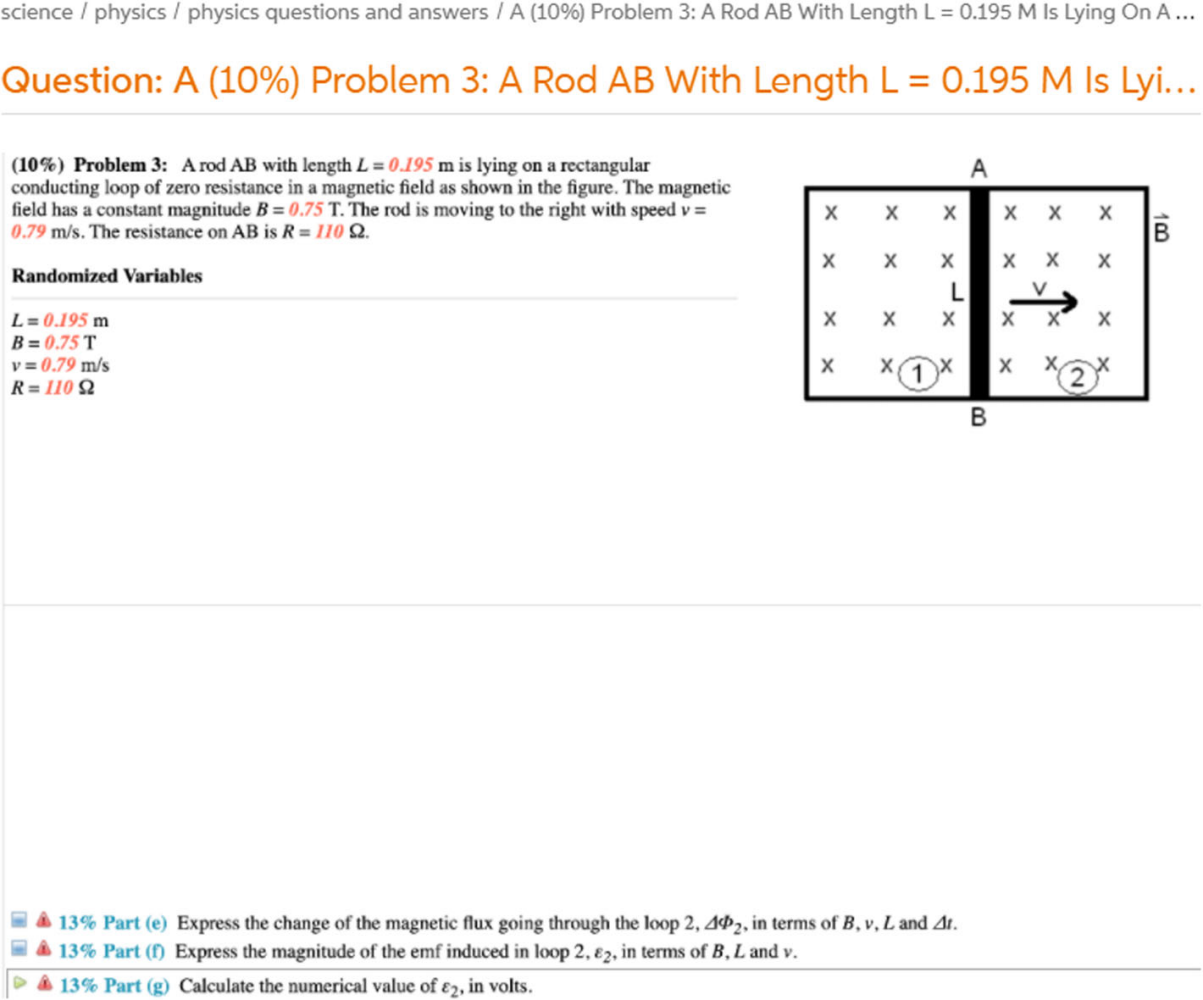
Example Question on Chegg likely from Online Exam

Many questions list a number of available marks on them. Others appear to contain randomised variables. These are indicators that the questions are likely from online exams. Some questions contain both marks and randomised variables, as indicated by the Physics question example shown in Fig. 7.

**Fig. 8:**
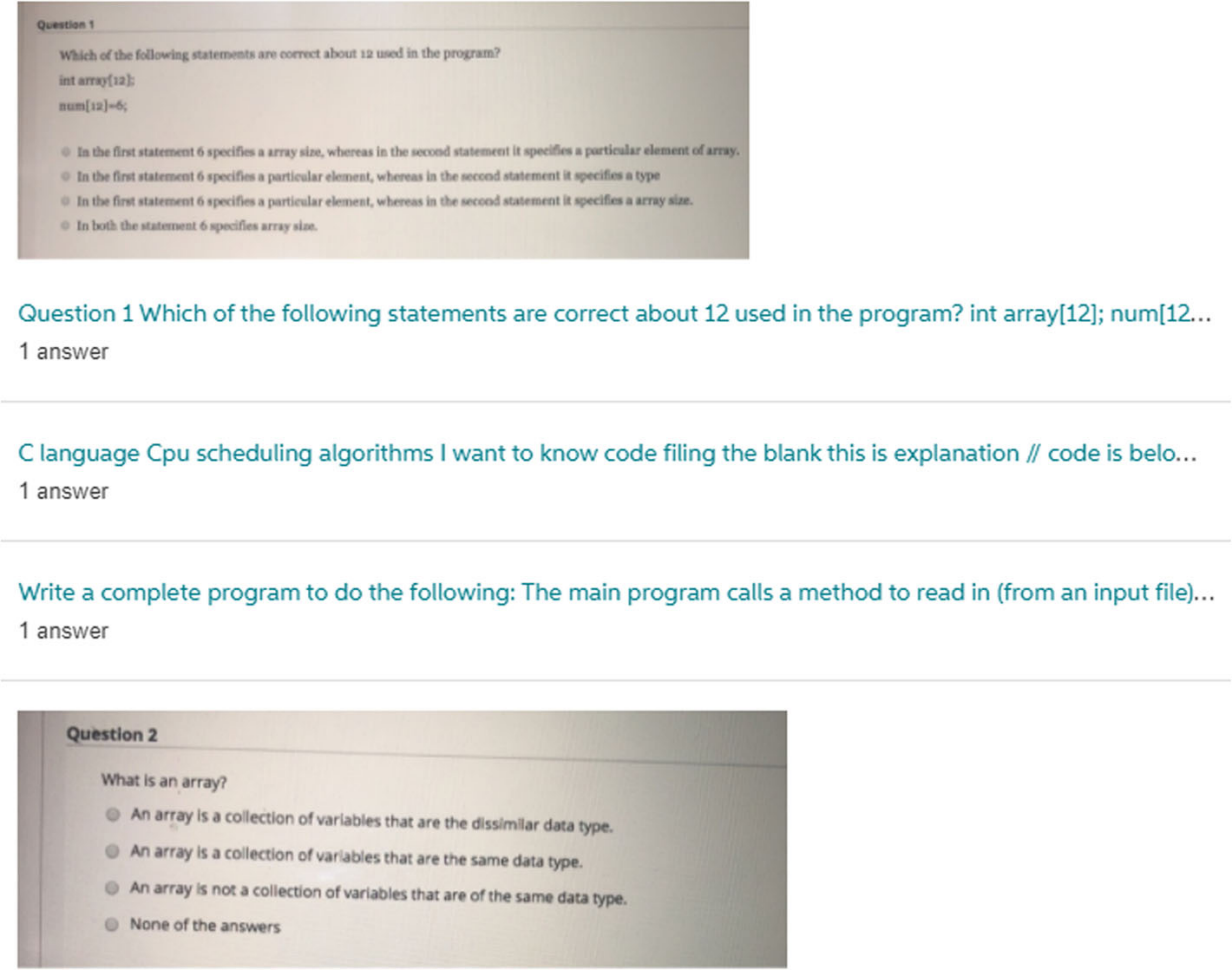
Question Series Example on Chegg likely from Online Exam

Students have also been observed posting a series of requests one after another, most often where the questions are multiple choice or short answer format. Figure 8 shows one such example for Computer Science, with a question sequence that extended far beyond the small sample shown. As occurs with many questions on Chegg, these take the form of low-quality images, which would make any form of automated processing of these difficult. Questions requested by other students were made in between these, demonstrating just how quickly requests appear on Chegg during peak periods.

An interesting consequence of the Covid-19 pandemic is that there are many questions on Chegg whose themes include Covid-19. A selection of terms such as COVID, pandemic and coronavirus were used as search terms across all questions on Chegg from all subjects. In every case, Chegg returned 500 results, which is the limit of the number of questions returned by a search. This indicates that students around the world are being set questions on Covid-19 and that many of them are finding their way onto file sharing sites for third parties to answer on their behalf.

## Conclusion

This paper has reviewed how a file sharing website popular with students is used to facilitate contract cheating. There are many assessed coursework and exam questions posted and answered every day. The data shows a substantial increase in the number of questions asked and answered in the period from April 2020 onwards, largely matching with the move online of teaching and assessment. Given the number of exam style questions, it appears highly likely that students are using this site as an easy way to breach academic integrity by obtaining outside help.

Using the fact that around 85% of questions asked are answered at least once, it is possible to approximate the number of questions answered each year. Based on Table 2, this suggests that about 2.6 million questions were answered out of the 3 million questions posted in 2018–2019, and around 4.8 million out of the 5.3 million questions in 2019–2020 were answered. This approximation covers only the five STEM subjects reviewed in the paper and not the other subjects from which Chegg also offers solutions to students. The number of questions asked and answered on Chegg each year is likely to be many times greater.

To have a successful and growing business and to deliver a return to its shareholders, Chegg depends on a continual growth in the number of subscribers it has. Based on the growth in the number of questions asked, this also suggests an increase in subscribers and thus the number of students who have access to the answers.

The apparent growth in student cheating also matches an increase in the market value of Chegg. Table 5 contains the value of shares at the beginning of each month, retrieved from Google after searching ‘Chegg valuation’. Since not all months have an entry for the first day of the month, the value has been obtained from the first available date. Table 5 indicates that between October 2019 and May 2020 there was an increase of no more than $14 USD, but then the share price increased by over $20 USD in the month following May.

**Table 5:** Chegg share price, by month

	Price of a Chegg share in USD
1 October 2019	29.21
1 November 2019	30.75
2 December 2019	38.50
2 January 2020	39.03
4 February 2020	41.75
2 March 2020	40.75
1 April 2020	34.13
1 May 2020	42.36
1 June 2020	62.43
1 July 2020	68.08
3 August 2020	85.93
1 September 2020	76.58

In an interview, Chegg’s chief executive mentioned that they noticed a “sustainable growth” of the website starting from 15 March 2020 (Gelles 2020). This further corresponds to the data presented in this paper and the increase in the market value of Chegg and suggests that contract cheating may have been a factor here.

Chegg does claim to have an Honour Code in place. In the Honour Code, it is stated that Chegg does not support fraud, cheating or breaches of copyright and suggests that materials may be removed or user accounts terminated if academic institutions contact them to open an investigation. There is little evidence that account termination has happened. In addition, it appears that there is nothing to stop students posting questions online and receiving answers within the time frame of an exam. There does not seem to be any manual approval of requests or periods of delay before questions are put live to be answered. People, including Chegg certified experts, appear to be ready and waiting to answer questions as they are asked. This seems to provide evidence that the Chegg Honour Code system is not working based on the volume of requests for assessed work that have been observed.

Some individuals have stated that requests can be made to Chegg if their copyrighted teaching or assessment material is found online (Murdoch 2020; Reddit 2020). This may include the details of accounts who have accessed the material, including their name, email address and institution. However, there has also been discussion online that the process is onerous, requires senior authorisation from universities and that students are given the opportunity to remove their accounts prior to the investigation so that no information can be transferred. This suggests that Chegg do not really want to eliminate contract cheating.

Future action in this area is necessary. This includes awareness raising with both staff and students. Using contract cheating services is not victimless and this has to be communicated. Similarly, the messaging needs to provide consistent and clear advice that there are benefits to working with academic integrity and there are risks involved when breaching it. Staff should be encouraged to monitor file sharing sites for their assessments, but this can be difficult when questions are posted as images rather than text. Some form of automated monitoring, with immediate reporting to instructors if their assessments or exam questions appear to be found online, would be a useful development.

Further research data could be collected from Chegg, covering more subjects over a longer time period. This investigation has focused only on STEM subjects, which typically have many questions that are mathematical or scientific in nature. This may make this area easier for contract cheating providers to give quick answers for these subjects than it would be for those that are more text based or descriptive. In addition, content analysis of the questions posted would also be useful, but that would most likely require an automated or machine learning based approach. Although it is the market leader, Chegg is not the only file sharing site. It would be instructive to see if the trends identified here extend more widely.

Academic integrity breaches are happening and, as the data presented in this paper has demonstrated, such breaches are becoming more common as a result of Covid-19. These breaches do require a continued reconsideration of teaching and assessment methods. Even if face-to-face teaching returns, it is unlikely that this will ever now consistently take the same format it did prior to the pandemic. The genie is well and truly out of the bottle and there is no way to put the stopper back in. The entire academic integrity community, including but not limited to staff, students, academic institutions, quality bodies and commercial providers alike, needs to be ready and prepared to act.

## Data Availability

An anonymised version of the quantitative data analyzed during the current study is available from the corresponding author on reasonable request.

## Abbreviations

ICAI: International Center for Academic Integrity
IDOA: International Day of Action against Contract Cheating
STEM: Science, Technology, Engineering and Mathematics
USD: United States Dollars
